# Should oral gavage be abandoned in toxicity testing of endocrine disruptors?

**DOI:** 10.1186/1476-069X-13-46

**Published:** 2014-06-25

**Authors:** Laura N Vandenberg, Wade V Welshons, Frederick S vom Saal, Pierre-Louis Toutain, John Peterson Myers

**Affiliations:** 1 Division of Environmental Health Sciences, University of Massachusetts – Amherst, School of Public Health, Amherst, MA 01003, USA; 2 Department of Biomedical Sciences, University of Missouri-Columbia, Columbia, MO, USA; 3 Division of Biological Sciences, University of Missouri-Columbia, Columbia, MO, USA; 4 Université de Toulouse, INPT, ENVT, UPS, UMR1331, F- 31062 Toulouse, France; 5 INRA, UMR1331, Toxalim, Research Centre in Food Toxicology, F-31027 Toulouse, France; 6 Environmental Health Sciences, 421 Park St., Charlottesville, VA 22902, USA

**Keywords:** BPA, Complications, Corticosterone, Glucuronidation, Hormone, Pharmacokinetic, Sublingual

## Abstract

For decades, hazard assessments for environmental chemicals have used intra-gastric gavage to assess the effects of ‘oral’ exposures. It is now widely used – and in some cases required – by US federal agencies to assess potential toxicity of endocrine disrupting chemicals (EDCs). In this review we enumerate several reasons why gavage is not appropriate for the assessment of EDCs using bisphenol A (BPA) as a main example. First, whereas human dietary exposures interact with the oral mucosa, gavage exposures avoid these interactions, leading to dramatic differences in absorption, bioavailability and metabolism with implications for toxicokinetic assumptions and models. Additionally, there are well acknowledged complications associated with gavage, such as perforation of the esophagus that diminish its value in toxicological experiments. Finally, the gavage protocol itself can induce stress responses by the endocrine system and confound the assessment of EDCs. These serious flaws have not been taken into account in interpreting results of EDC research. We propose the exploration of alternatives to mimic human exposures when there are multiple exposure routes/sources and when exposures are chronic. We conclude that gavage may be preferred over other routes for some environmental chemicals in some circumstances, but it does not appropriately model human dietary exposures for many chemicals. Because it avoids exposure pathways, is stressful, and thus interferes with endocrine responses, gavage should be abandoned as the default route of administration for hazard assessments of EDCs.

## Background

The realization that a large number of chemicals used in products disrupt endocrine function, referred to as endocrine disrupting chemicals (EDCs), has created significant challenges for the approaches used in hazard assessments of environmental chemicals. Knowledge generated in the last several decades about EDCs questions many assumptions used in chemical risk assessments. This has led to a number of published studies disputing current hazard and risk assessment approaches and assumptions for studies of EDCs [1-5].

In this review we examine a method commonly used in toxicology studies, intra-gastric gavage, to deliver chemicals to subjects in a controlled manner. Intra-gastric gavage involves the insertion of a tube into the mouth, the sliding of this tube through the esophagus, and the deposition of the compound directly into the stomach [6]. This method has been widely used to test potential hazards of EDCs because it is thought to allow precise control of both the dose and timing of treatment. Two comparable delivery methods used in humans involve enclosing the chemical in pills that only release their contents in the stomach and the delivery of nutrients directly to the stomach of patients (enteral dosing) via a feeding tube.

We specifically address recent studies that challenge the use of gavage for the study of EDCs using bisphenol A (BPA) as a model EDC. We chose BPA because human exposures are widespread [7], low doses have been linked to adverse effects in laboratory animals [8,9], exposures are associated with a wide range of human diseases [10], and there remain unanswered questions about how best to model routes and sources of exposure [11]. Although we chose to focus on BPA, the issue of a lack of detailed understanding of all potential routes of exposure applies to many chemicals used in a wide range of products [12].

We also review a number of studies suggesting that gavage does not appropriately model dietary chemical exposures and what the consequences might be for the interpretation of toxicokinetic studies. We discuss how gavage can induce stress responses that may interfere with hazard assessments of chemicals that have endocrine modes of action. We conclude by identifying other methods of chemical administration and address how to best mimic exposures experienced by humans.

### Gavage does not model human dietary exposures

When exposures occur via the diet, the chemical can interact with numerous surfaces in the oral cavity including the buccal, sublingual, gingival, palatal and labial mucosa [13]. Each of these surfaces is fed by a rich blood supply, and the epithelia within the oral mucosa are 4 to 4000-times more penetrable than skin [13]. Chemical absorption occurs efficiently via these surfaces, leading to rapid transport to the arteries delivering the chemical to tissues. Importantly, chemicals that are absorbed via this route evade a sequential first-pass metabolism first by the gut wall and then by the liver. For example, the gut wall first-pass effect is relevant in rats for BPA, but not in humans [14], whereas hepatic first-pass metabolism is most important in humans [15,16]. When chemicals that are absorbed in the mouth evade first-pass metabolism, they are then bioavailable at higher levels relative to chemicals absorbed from the gut that are transported directly to the liver via the mesenteric vessels.

The toxicokinetic profiles of several chemicals have been shown to differ substantially in animals treated via gavage versus administration via other oral routes. In one example, rodents were administered donepezil, a pharmaceutical used in the treatment of Alzheimer’s disease, either via gavage or in a solution consumed from a syringe [17]. Both blood and brain concentrations were lower when the drug was administered via gavage compared to when it was swallowed, differences the authors attributed to absorption via the buccal and/or sublingual surfaces. Steroid hormone absorption is more rapid and leads to higher blood concentrations when administered sublingually than when ingested via a capsule or pill [18]. Gavage versus dosed feed administration of benzyl acetate affected the carcinogenic response by this chemical [19]. Finally, for Sulindac, a non-steroidal anti-inflammatory, oral gavage resulted in higher peak and lower trough concentrations in plasma and mammary tissue, and had a greater effect on prostaglandin E2 levels than the corresponding dietary dosing [20].

In a recent study, circulating BPA concentrations were examined in dogs administered BPA via the sublingual mucosa or via traditional gavage methods [15]. Following sublingual exposure, BPA entered into circulation largely in an unconjugated form, consistent with a pathway that evades hepatic first-pass metabolism. This contrasted with the results of gavage administration in the same study, in which over 99% of BPA reaching the systemic circulation was in a conjugated (glucuronidated) form, as would be expected when a compound undergoes extensive first-pass metabolism [15,21,22]. An interesting additional comparison is provided by studies in which BPA was administered to rhesus monkeys via gavage [23] and less than 1% of administered BPA was bioavailable (unconjugated) in blood; in contrast, BPA fed in a piece of fruit resulted in over 7% of administered BPA being bioavailable in blood [16,24].

A human *de facto* variant of gavage involves delivery of the chemical in hard pills or gelatinous capsules. Like gavage, this form of administration prevents the chemical from interacting with the oral mucosa, leading to toxicokinetic parameters that are unlikely to reflect all of the human experience for a chemical that people are exposed to via an oral route. Unfortunately, the only experimental human BPA toxicokinetic study conducted to date delivered the chemical using a hard gelatin capsule [25], rendering their results irrelevant to real-life human exposures via the diet.

For chemicals like BPA found as contaminants in food and beverages, human exposures via dietary sources likely occur throughout the day in meals and snacks. Buccal absorption associated with discrete meals should generate a series of BPA peaks in the blood and then troughs during the inter-digestive period, rather than a low steady exposure expected following intestinal absorption. In addition, the amplitude of these intermittent bursts of BPA concentrations could be higher in arterial blood than those that can be measured peripherally [26]. Although there are protocols to employ the gavage method of administering chemicals to animals repeatedly during the day, handling animals and inserting a tube into their stomach multiple times daily is clearly problematic. Thus, studies aimed to assess the hazards of chemicals have used gavage protocols that typically rely on a single daily administration [see for example [27-29]]. These differences between real-world experience and experimental protocol in the timing and duration of exposures reduce the utility of gavage protocols for studies concerned with mimicking human dietary exposures. A study that directly compared the toxicokinetic profiles of BPA in mice exposed via gavage and those exposed in their feed revealed higher concentrations of unconjugated BPA in the blood of mice that consumed BPA via food versus those that were treated via gavage [30]. In fact, after adjusting for dose, the maximum serum concentration reached was 3-fold greater when BPA was consumed in feed vs. when administered via gavage. Note that the rodent mouth is more heavily keratinized compared to dog or primate [15], reducing but likely not eliminating oral absorption.

In sum, exposure by gavage or by swallowing a capsule may result in significant differences in toxicokinetics compared to an oral exposure that mimics the routes and timing of exposure to chemicals consumed in food or beverages. There are two principle reasons: gavage avoids absorption by surfaces in the oral cavity, and it typically involves only a single daily pulse (bolus). An analysis of data that showed that time since the last meal did not account for BPA levels in urine in the US population [31] suggested that gavage models [32,33] are likely not accounting for major sources and routes of BPA exposure.

### Why are these differences in toxicokinetic parameters important?

Calculating toxicokinetic parameters from data obtained in gavage and/or hard gelatin capsule experiments could lead to inaccurate conclusions about expected bioactive levels of EDCs in serum in the general population. For example, because gavage/capsule experiments show that over 99% of BPA absorbed in the gastrointestinal tract undergoes first pass metabolism, a recent study estimated that the amount of unconjugated BPA in human serum would be below the limit of detection of even the most sensitive analytical methods available [34]. This analysis assumes that no absorption occurs via the oral mucosa; yet as discussed above, oral absorption can be substantial and, in fact, not significantly different from exposure via IV injection [15]. The assumption that almost all BPA from oral exposures undergoes first-pass metabolism has been used to argue that any observed unconjugated BPA in human serum must be the result of contaminated lab procedures, and therefore has been used to dismiss dozens of biomonitoring studies that report unconjugated BPA in human serum samples [11].

A recent NIH-sponsored study assessed whether BPA could be measured accurately in human serum by multiple laboratories and whether there was contamination during blood collection [35]. The conclusion was that sources of contamination could be identified and eliminated, allowing contamination-free assays to be conducted [36]. Other studies have similarly reported that BPA contaminations can be eliminated during blood collection. For example, one study referred to BPA contamination as “an elusive laboratory challenge” [37] and another noted “a propensity to introduce artifactual aglycone BPA” into assays [23], yet both of these studies report that sources of BPA contamination could be identified and eliminated prior to collection and assay of serum samples. Thus, BPA contamination can be controlled when assaying human serum and urine samples. Suggestions that unconjugated BPA cannot be detected in human serum also imply that BPA should have no effects on human health at current levels of exposure [34]. Yet over sixty epidemiology studies show associations between BPA exposures and disease outcomes [10].

### Stress associated with gavage is problematic for endocrine endpoints

A large literature has examined the effects of gavage on stress. A meta-analysis of experiments designed to assess the effects of gavage (without assessing the effects of specific test chemicals) indicates that the process of gavaging animals induces rapid, pronounced and statistically significant effects on stress-related responses [38]. Individual studies have shown that gavage can increase the secretion of the stress-response hormone corticosterone in mouse feces, evidence that the hypothalamic-pituitary-adrenal axis is activated [39]. The effects on corticosterone levels can vary depending on the vehicle and volume of liquid used [40,41]. Other studies indicate that gavage affects cardiovascular endpoints including diastolic and systolic blood pressure and heart rate [38,42]. Interestingly, Okva and colleagues demonstrated that small volumes of gavage fluid have greater effects on heart parameters than larger volumes [42], an unexpected finding that challenges the assumption that stress responses occur only when animals are gavaged with inappropriately high volumes. In these studies, physiological differences were apparent at least one hour after the gavage protocol. This is likely to be of particular importance when studying pregnant females; acute maternal stress causes elevated serum corticosterone levels to persist for 12 hours [43], indicating that a significant portion of the day could be spent with elevated circulating stress hormones due to stress during pregnancy.

Stress has a significant impact on some of the major drug-metabolizing enzyme systems; different forms of stress were reported to either increase metabolic clearance [44] or decrease metabolic rate [45]. For example, stressed rats display significantly decreased blood levels of hexobarbital, pentobarbital and meprobamate, but not phenobarbital, after administration of these drugs [46]. The ability of stress to decrease drug levels in blood was shown to be dependent upon an intact pituitary-adrenal axis.

In an ongoing FDA-sponsored study that is examining the effects of BPA on physiological and behavioral endpoints, a wide range of BPA doses were administered daily by gavage first to pregnant rats and then to the offspring from birth for up to two years [35]. In the first published study from this experiment, BPA produced significant effects at the two lowest doses in the experiment (2.5 and 25 μg/kg/day) on expression of the genes for estrogen receptors alpha and beta, Esr1 and Esr2, in the brains of neonates [47]. The authors also reported that the gavage protocol alone altered these endpoints compared to pups produced from mothers that had not been gavaged but had been subjected to all other handling and restraint procedures; restraint is commonly used as a stress-inducing procedure in rats [48]. Cao et al. noted that gavage may confound the study of BPA and other EDCs [47], writing, “Interpreting the BPA and [ethinyl estradiol] results in regions where there were statistically significant differences in [estrogen receptor] expression between the naïve and vehicle controls requires caution because the BPA- or [ethinyl estradiol]-related effects on expression may have been influenced by prenatal stress (from gavage)”. Thus, there is evidence that restraining and gavaging animals induces stress responses that can increase the probability of false negative or false positive findings if appropriate controls are not employed in endocrine disruption research.

Unfortunately, in the next phase of the FDA study, the only control animals not treated with BPA are being subjected to both restraint and daily gavage, so the control animals will be subjected to significant multiple sources of stress on a daily basis beginning during fetal life and then continuing from birth, for as long as 2 years. As noted by Cao and colleagues [47], this experimental design will impair the ability to distinguish the effects of the compound from the effects of stress.

Roberts et al. reported that gavage can also induce significant levels of apoptosis in the liver, a response with obvious physiological consequences [49]. Because this study was not initially designed to test the effects of gavage itself, the authors concluded that without the use of appropriate control groups (the inclusion of both untreated controls and vehicle gavage controls), completely different conclusions could have been made about the effects of their test chemical on the liver. Roberts et al. also demonstrated that the effects of gavage on liver apoptosis can be masked by co-treatment with compounds that act as liver hyperplastic agents, increasing concerns that gavage can confound or alter the results of studies designed to test the health effects of pharmaceuticals or xenobiotics. These concerns were again raised by Okva et al. [42], who noted that “[p]hysiological, metabolic, endocrine and behavioural changes can be attributed to stress, and [intragastric]-gavaging procedures as such may interfere with the evaluation of novel drugs administered by this route.”

Collectively, this evidence has wide-reaching implications. Thousands of studies performed by regulatory agencies, academic researchers and industry labs have used gavage to assess thousands of compounds, including EDCs. Data showing that gavage activates the stress response system indicate that these time-consuming and costly studies cannot be interpreted as showing effects (or no effect) of any chemical due to the confounding factor of stress; at best, positive findings can only be interpreted as due to an interaction between stress and chemical exposure. More problematic from a public health perspective is the interpretation of results that do not show a significant effect (i.e. negative results), since assumptions regarding the absence of hazard may be based on masking of an effect by the stress associated with gavage. Multiple controls are required to separate these interacting factors, and studies that do not include all of the appropriate controls cannot be easily interpreted.

### Acknowledged complications with gavage

The issues we have raised here are largely but not completely unacknowledged in the literature and are compounded by other issues also widely recognized. For example, a common complication with gavage involves perforation of the esophagus or stomach [41]. Additional complications involve the accidental introduction of fluids into the trachea or lungs, asphyxia, inflammation, weight loss, hemorrhage, and reflux [41,50]. Other studies report high frequencies of morbidity and mortality due to the use of gavage; several report rates greater than 50% [38]. Numerous factors can influence the success of gavage, including the level of experience of the technician, size and type of probe used, volume administered, repetitive dosing and vehicle used [42]. Because of the relatively high frequency of gavage-related complications and the need for highly trained animal care staff, some animal welfare groups propose that alternative dosing methods be used whenever possible [6].

Gavage has been used in many hazard assessments because this method is thought to allow precise control of both the dose and timing of treatment. Yet, recent studies have revealed that this assumption may not be accurate. In a recent study using ‘Good Laboratory Practices’ (GLP), scientists from the FDA reported substantial BPA contamination (including serum concentrations of BPA-metabolites consistent with uncontrolled exposures of up to 80 μg BPA/kg/day) in two sets of negative controls: those that were administered an oil vehicle, and those that were not gavaged with any compound [51,52]. Unfortunately, the source of the contamination could not be identified, so it cannot be determined what role the gavage method itself played in this inadvertent exposure. Importantly, these results suggest that the positive aspects of using gavage (i.e. precise control of exposures) may over-estimate the value of this exposure route, and thus other large GLP-compliant studies that have used gavage but have not measured BPA and BPA metabolites in serum to evaluate actual contamination should be examined with more caution (for example, [53,54]).

### Should endocrine disruptor screening assays use gavage?

The US EPA’s Endocrine Disruptor Screening Program states that gavage delivery is preferred in its Tier 1 testing protocols [55]. All of the studies included in their validation of two protocols, the Hershberger assay and the TG-407 28-day oral toxicity study, solely used the gavage method [56]. Similarly, the NTP’s developmental and reproductive screening assays are expected to use the most relevant route of exposure for the chemical of interest, but note that when conducting toxicokinetic studies, “[i]f feed or drinking water will be the exposure route in the toxicology study, gavage exposure should also be included to estimate the absorption parameter(s)” [57].

Data from toxicokinetic studies and data from studies investigating the effects of gavage itself do not support the use of gavage in the testing of putative EDCs. Clearly, toxicokinetic profiles can differ when exposures occur via gavage versus other routes of exposure [15]. Additionally, the stressful nature of the gavage method can alter function of the hypothalamic-pituitary-adrenal axis [39] and could confound the assessment of chemicals that interfere with hormones that act on this axis. Because the endocrine system has complex positive- and negative-feedback loops, the effects of a stressful event may not be limited to endpoints associated with the hypothalamic-pituitary-adrenal axis [58] – challenging the use of gavage for the assessment of any endocrine-responsive endpoint. As noted elsewhere, the gastrointestinal tract “is the largest endocrine organ system in the body, and may secrete more hormones than all other organ systems combined” [59]. Therefore, it is essential to understand the implications of the administration of EDCs to the gastrointestinal tract, for example by requiring the inclusion of both non-gavaged controls as well as vehicle-only gavage controls.

### Other options for chemical administration

For hypothesis testing, route of exposure may not be of central importance, but for hazard assessment, risk assessors typically require that studies use a route of exposure that is deemed “relevant” to humans [57]. There are exceptions to this requirement: the FDA requires that all animal studies used in hazard assessments employ oral exposure routes, even if non-oral routes occur or are expected; an example is diethyl-phthalate (DEP), a compound used in cosmetics with dermal and inhalatory human exposure routes that has been tested in animals via oral administration [60].

All dosing methods have pros and cons that must be considered in the design of a study, bearing in mind how the data will be used. Most of this review has focused on limitations to the gavage method, particularly because we have not seen these issues acknowledged in a regulatory context, and because recent studies suggest that the use of gavage may interfere with the study of EDCs [47]. Yet, EDCs and pharmaceuticals have also been administered by milling the compound of interest into feed, dissolving it in drinking water, feeding animals from a pipette, or adding the compound to a wafer or other food and allowing the animal to consume it. Although the FDA and other regulatory or advisory panels have clearly given priority to studies that use oral and gastric exposures [61,62], it is important to note that there are alternatives, including Silastic implants and osmotic pumps. These implanted devices are of particular interest in BPA studies because they can provide constant exposures to low doses that produce serum concentrations that approximate those found in humans [16,63,64]. These routes of exposure may be relevant also because there are important and significant non-oral sources of BPA exposure [11,65]. The issue of stress during the placement of implanted devices, and also the possibility of chronic inflammation that can alter disposition of many substances and hormones, however, requires further attention.

### Conclusions: how well do exposure protocols in experimental studies mimic human exposures?

For a chemical like BPA, the majority of exposures are currently thought to come from food, but by no means does this mean that gavage exposures adequately model human dietary exposures, nor should this prediction eliminate from consideration non-dietary sources [65]; the European Food Safety Authority recently acknowledged that there was “uncertainty” regarding the degree to which thermal paper contributed to BPA exposure in the general population [66]. In the study of chemicals with multiple routes and chronic exposures, which method of administration appropriately mimics this situation? At one end of the spectrum, it has been proposed that as long as a method produces circulating blood concentrations that are within the range of what has been measured in humans, the study should be considered relevant, regardless of how the chemical was administered [2]. At the other end of the spectrum is the FDA’s stance that only oral exposures, preferably by gavage, are relevant, even if actual human exposures occur via other routes.

We conclude that the use of gavage administration as the recommended approach may be inappropriate in the study of EDCs because it can confound studies by inducing substantial stress in animals, thus altering all endocrine and non-endocrine responses associated with a regular meal. Gavage also avoids oral absorptions that are part of human dietary exposure. Although there are limitations to all methods of dosing animals, the flaws associated with gavage are so severe for some substances, especially those subjected to a large first-pass effect, that this route of exposure should be abandoned for the study of EDCs. For some chemicals, there may be reasons why gavage is preferred over other routes of administration, but this method should not be employed using the rationale that it ‘appropriately models human dietary exposures’; the data do not support this assumption.

## Abbreviations

BPA: Bisphenol A; DEP: Diethyl-phthalate; EDC: Endocrine disrupting chemical; EPA: Environmental Protection Agency; FDA: Food and Drug Administration; GLP: Good Laboratory Practices; NTP: National Toxicology Program; TCDD: 2,3,7,8-tetrachlorobenzo-*p*-dioxin.

## Competing interests

WVW, P-LT and JPM declare no conflicts of interest. LNV provided expert testimony in a civil case involving a product that might contain EDCs. FVS wrote a report for attorneys involved in product labeling litigation.

## Authors’ contributions

LNV, WVW, FSvS, P-LT and JPM wrote the paper. All authors read and approved the final manuscript.
